# Association between physical activity and mortality in postmenopausal women: evidence from NHANES 2007–2018

**DOI:** 10.1186/s12905-026-04315-3

**Published:** 2026-02-09

**Authors:** Baixiang Zhang, Mu Yang, Gareth Ambler, Shuangfang Fang, Qilin Yuan, Yixian Zhang, Nan Liu, Houwei Du

**Affiliations:** 1 Department of Rehabilitation, Longyan First Affiliated Hospital of Fujian Medical University, Longyan, 364099 China; 2 Stroke Research Center, Department of Neurology, Fujian Medical University Union Hospital, Fuzhou, 350001 China; 3 Institute of Clinical Neurology, Fujian Medical University, Fuzhou, 350001 China; 4 Department of Statistical Science, University College London, WC1E 6BT London, UK; 5 Department of Rehabilitation, Fujian Medical University Union Hospital, Fuzhou, 350001 China

**Keywords:** Physical activity, Postmenopausal women, Mortality

## Abstract

**Background:**

Whether physical activity (PA) levels relate to mortality in postmenopausal women remains not well understood.

**Methods:**

We analyzed 5,880 postmenopausal women from NHANES 2007–2018 (median follow-up:77 months). PA—including total PA (TPA), leisure-time PA (LTPA), and occupational PA (OPA)—was assessed using the Global Physical Activity Questionnaire and expressed as metabolic equivalent of task (MET)-minutes/week. TPA was classified as no, insufficiently active (< 600), or sufficiently active (≥ 600), while LTPA and OPA were classified as no, low (< 600), or high (≥ 600). Weighted proportional hazards Cox models and restricted cubic spline (RCS) analyses examined associations with all-cause, cardiovascular disease (CVD), and non-CVD mortality.

**Results:**

During follow-up, 718 deaths occurred (215 CVD, 503 non-CVD). Compared with no PA, insufficiently and sufficiently active TPA were associated with reduced risks of all-cause, CVD, and non-CVD mortality; both low and high LTPA with reduced risks of all-cause and non-CVD mortality; and low OPA with reduced risks of all-cause and non-CVD mortality. RCS analyses showed linear inverse associations for TPA and non-linear inverse associations for LTPA with all-cause and non-CVD mortality, while OPA showed no significant associations. Tests for trend were significant for TPA in relation to all-cause and non-CVD mortality, and for LTPA across all three outcomes. Sensitivity analyses excluding early deaths yielded similar results.

**Conclusions:**

Higher TPA and LTPA were associated with reduced risks of all-cause and non-CVD mortality among postmenopausal women. Promoting adequate PA, particularly moving from inactivity to modest levels, may be an effective strategy to support healthy aging in this population.

**Supplementary Information:**

The online version contains supplementary material available at 10.1186/s12905-026-04315-3.

## Background

Substantial evidence has shown that physical activity (PA) is associated with lower risks of all-cause and cause-specific mortality in general population [1, 2]. The U.S. Physical Activity Guidelines recommend adults engage in at least 150–300 min of moderate-intensity or 75–150 min of vigorous-intensity PA per week, which, based on the World Health Organization (WHO) conversion, corresponds to approximately 600–1200 metabolic equivalent of task (MET) minutes [3, 4]. Moreover, additional health benefits are conferred by exceeding these minimum levels of activity [5, 6]. Despite broad consensus on the health-promoting effects of PA in the general adult population, its impact may differ across subgroups with unique physiological profiles.

Postmenopausal women represent a particularly vulnerable population, undergoing substantial physiological and hormonal changes following the cessation of ovarian estrogen production [7]. Postmenopause can be classified into natural menopause (gradual hormonal transition) and surgical menopause (abrupt hormonal transition), such as hysterectomy with bilateral salpingo-oophorectomy [8]. This hormonal change contributes to an elevated risk of cardiovascular disease (CVD), osteoporosis, central adiposity, insulin resistance, and metabolic syndrome [9–12], which may shorten life expectancy and increase risks of mortality. The Iowa Women’s Health Study showed that moderate leisure-time physical activity (LTPA) once per week was associated with reduced all-cause mortality, and higher levels of PA conferred greater protection [13]. However, not all types of PA exert the same health effects. The Copenhagen General Population Study showed that LTPA was linked to lower all-cause and CVD mortality, whereas occupational physical activity (OPA) was paradoxically associated with higher mortality—a phenomenon termed the “physical activity paradox” [14].

To our knowledge, evidence regarding different domains of PA and mortality among postmenopausal women remains limited. To address this gap, we used data from the National Health and Nutrition Examination Survey (NHANES), a nationally representative U.S. dataset with long-term mortality follow-up, to examine the association between PA levels and mortality outcomes among postmenopausal women.

## Methods

### Study design and population

The NHANES is a nationally representative survey of the civilian, non-institutionalized U.S. population, conducted by the National Center for Health Statistics (NCHS) at the Centers for Disease Control and Prevention (CDC) [15]. All NHANES participants provided written informed consent at the time of survey enrollment, and the protocols were approved by the National Center for Health Statistics Research Ethics Review Board [16].

This study analyzed data from the NHANES 2007–2018 cycles, which initially included 30,213 female participants. Menopausal status was determined using two items from the self-reported reproductive health questionnaire: (1) Have you had at least one menstrual period in the past 12 months?” and (2) “What is the main reason for not having a menstrual period in the past 12 months?” Participants were considered postmenopausal if they answered “No” to the first question and “Menopause/Hysterectomy” to the second. Inclusion criteria were: (1) female menopausal participants; (2) participants with complete information about PA and survival. Exclusion criteria were as follows: (1) age < 20 years; (2) reporting regular menstrual cycles or missing menstrual history; (3) menstrual irregularities due to causes other than menopause/hysterectomy or for unknown reasons; (4) missing covariate data.

### Physical activity assessment

PA was assessed using the Global Physical Activity Questionnaire (GPAQ). Each participant’s PA was converted to MET minutes/week based on the reference values provided by NHANES. According to the American Physical Activity Guidelines, adults are recommended to engage in at least 150 min of moderate-intensity physical activity per week or 75 min of vigorous-intensity physical activity per week, which is equivalent to 600 MET-minutes per week [3]. In this study, total PA (TPA) was categorized into no TPA, insufficiently active TPA (1–599 MET-min/ week) and sufficiently active TPA (≥ 600 MET-min/week). Leisure-time PA (LTPA) and occupational PA (OPA) were categorized into No PA (0 MET-min/week), low PA (1-599 MET-min/week) and high PA (≥ 600 MET-min/week) separately [17]. We also categorized PA into finer strata (0, 1-599, 600–1199, 1200–1799, 1800–2999, 3000–5999, and ≥ 6000 MET-min/week) to reflect multiples of the guidelines PA recommendations, ranging 0 to up to ≥ 10 times the recommended levels. These thresholds were originally developed for LTPA [6]. For LTPA, due to the limited number of participants in the upper categories (3000–5999 and ≥ 6000 MET-min/week), these groups were collapsed into one category (≥ 3000 MET-min/week).

### Survival outcome

Mortality data were obtained from the CDC and linked to the NHANES database using unique participant identifiers. Death status was ascertained through December 31, 2019, based on information available at the CDC’s data linkage portal (https://www.cdc.gov/nchs/data-linkage/mortality.htm). Deaths were classified using ICD-10 codes: CVD mortality included diseases of the heart (I00–I09, I11, I13, I20–I51) and cerebrovascular diseases (I60–I69). Non-CVD mortality comprised all other causes, mainly malignant neoplasms, chronic lower respiratory diseases, unintentional injuries, Alzheimer’s disease, diabetes mellitus, influenza and pneumonia, nephritis and related disorders, and other residual causes.

### Covariates

Our study accounted for a range of clinical covariates to minimize their potential confounding effects on the association between PA and mortality. These covariates included age, age at menopause, race/ethnicity, body mass index (BMI), education level, poverty-to-income ratio (PIR), marital status, alcohol consumption, smoking status, congestive heart failure, coronary heart disease, stroke, hypertension, diabetes, hyperlipidemia, menopausal hormone therapy (MHT), depression and oophorectomy history.

Age at menopause was obtained by the response to the question “Age at last menstrual period. ”. Race/ethnicity was categorized as Mexican American, other Hispanic, non-Hispanic White, non-Hispanic Black or other race; education level was classified as less than 9th grade, 9-11th grade, high school graduate (or GED), some college (or AA degree) and college graduate (or above); PIR was classified as PIR ≤ 100%, 100%< PIR < 300%, 300%≤PIR < 500% and PIR ≥ 500% [18]. Marital status was divided into two categories: married (or living with partner) and not married (nor living with a partner).

Alcohol consumption was based on the frequency the participant drinks. Participants who drink 12 or more times a year were classified as drinkers, and participants who drinks less than 12 times a year were classified as non-drinkers [19]. Smoking status was determined by the response to the question “Have you ever smoked at least 100 cigarettes in your lifetime?” Participants who answered “yes” (≥ 100 cigarettes) were classified as smokers, and those who answered “no” (< 100 cigarettes) were classified as non-smokers [19]. Congestive heart failure was obtained by the response to the question “Someone ever told you had congestive heart failure?”. Participants who answered “yes” were classified as having congestive heart failure, and those who answered “no” were classified as not having congestive heart failure [20]. Coronary heart disease, stroke, hypertension, diabetes, and hyperlipidemia were obtained by similar questions. MHT was obtained by the response to the question “Have you ever used female hormones such as estrogen and progesterone” [21, 22]. Depression status was determined using the Patient Health Questionnaire-9, with a score of 10 or higher indicating depression [19]. Oophorectomy history was obtained by the response to the question “Have you had both of ovaries removed”.

### Statistical analysis

All statistical analyses accounted for the complex survey design to ensure nationally representative estimates of the U.S. population. For the combined 2007–2018 data, the Mobile Examination Center (MEC) examination sample weights (WTMEC2YR) were divided by 6 to create appropriate 12-year weights, as recommended by the NHANES analytic guidelines. Continuous variables were summarized as means with standard deviations if normally distributed, and medians with interquartile ranges (IQR) if not normally distributed. Categorical variables were presented as absolute counts with proportions. Kruskal-Wallis test and chi-squared tests were used to compare differences between groups. Restricted cubic spline (RCS) models were used to explore the potential nonlinear associations of continuous PA and mortality outcomes, adjusting for all potential covariates. To improve interpretability, RCS plots were truncated at 10,000 MET-min/week, as values above this threshold represented less than 4.0% of the population. Kaplan-Meier curves for all-cause/CVD/non-CVD mortality were plotted during follow-up time (months) according to the PA levels. Cox proportional hazards regression models were used to calculate the Hazard Ratio (HR) with 95% confidence interval (CI) for the association of all-cause/CVD/non-CVD mortality with PA. The proportional hazards assumption was tested using Schoenfeld residuals, and no substantial violations were observed. Three models were used: Model 1 adjusted for age, age at menopause and race/ethnicity; Model 2 further adjusted for BMI, PIR, education level, marital status, alcohol consumption, and smoking status; and the fully adjusted Model 3 further adjusted for congestive heart failure, coronary heart disease, stroke, hypertension, diabetes, hyperlipidemia, MHT, depression and oophorectomy history [23–25]. A directed acyclic graph was constructed to illustrate the assumed causal relationships and to support the appropriateness of the selected adjustment set (Figure S1). In addition, to account for competing risks between CVD and non-CVD mortality, we conducted exploratory Fine–Gray sub-distribution hazard analyses in three-category exposure groups (No PA, Low PA, and High PA).

Two sensitivity analyses were performed to evaluate the robustness of the primary findings: (1) excluding those who died within the first 2-year follow-up; (2) in a complete dataset after multiple imputation for missing covariates (the numbers of missing values for key covariates were as follows: age at menopause [*n* = 374], education level [*n* = 14], BMI [*n* = 108], PIR [*n* = 793], marital status [*n* = 4], alcohol consumption [*n* = 217], smoking status [*n* = 5], congestive heart failure [*n* = 21], coronary heart disease [*n* = 31], stroke [*n* = 13], diabetes [*n* = 6], hypertension [*n* = 8], hyperlipidemia [*n* = 366], MHT [*n* = 56], depression [*n* = 136] and oophorectomy history [*n* = 168], and five imputed datasets were generated and used for subsequent analyses). All statistical analyses were done using R (version 4.4.1), with a two-sided P value < 0.05 considered statistically significant.

## Results

### Baseline characteristics of participants

**Fig. 1:**
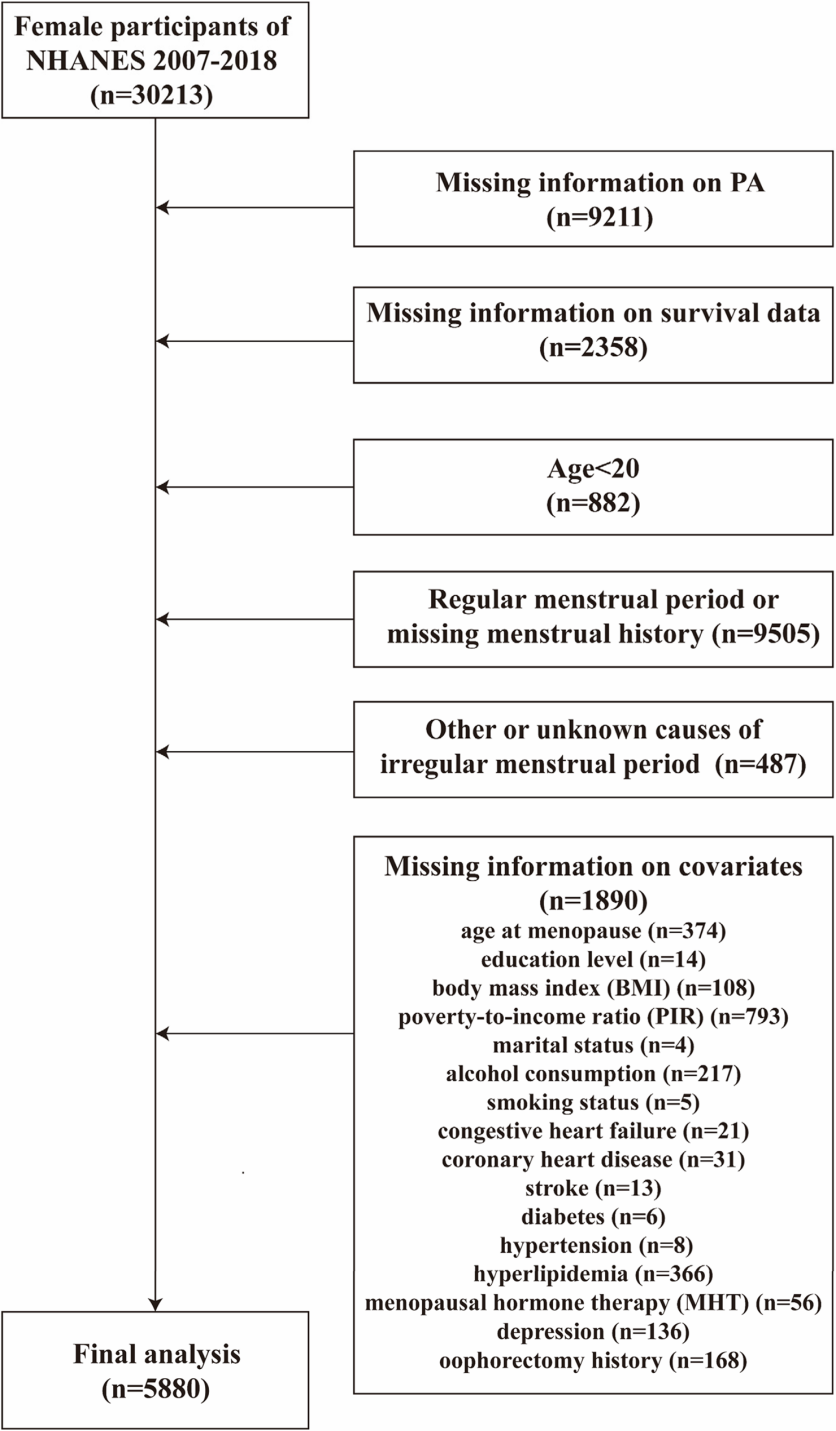
Flowchart of sample selection. Abbreviations: NHANES: National Health and Nutrition Examination Survey; PA: Physical activity

**Fig. 2:**
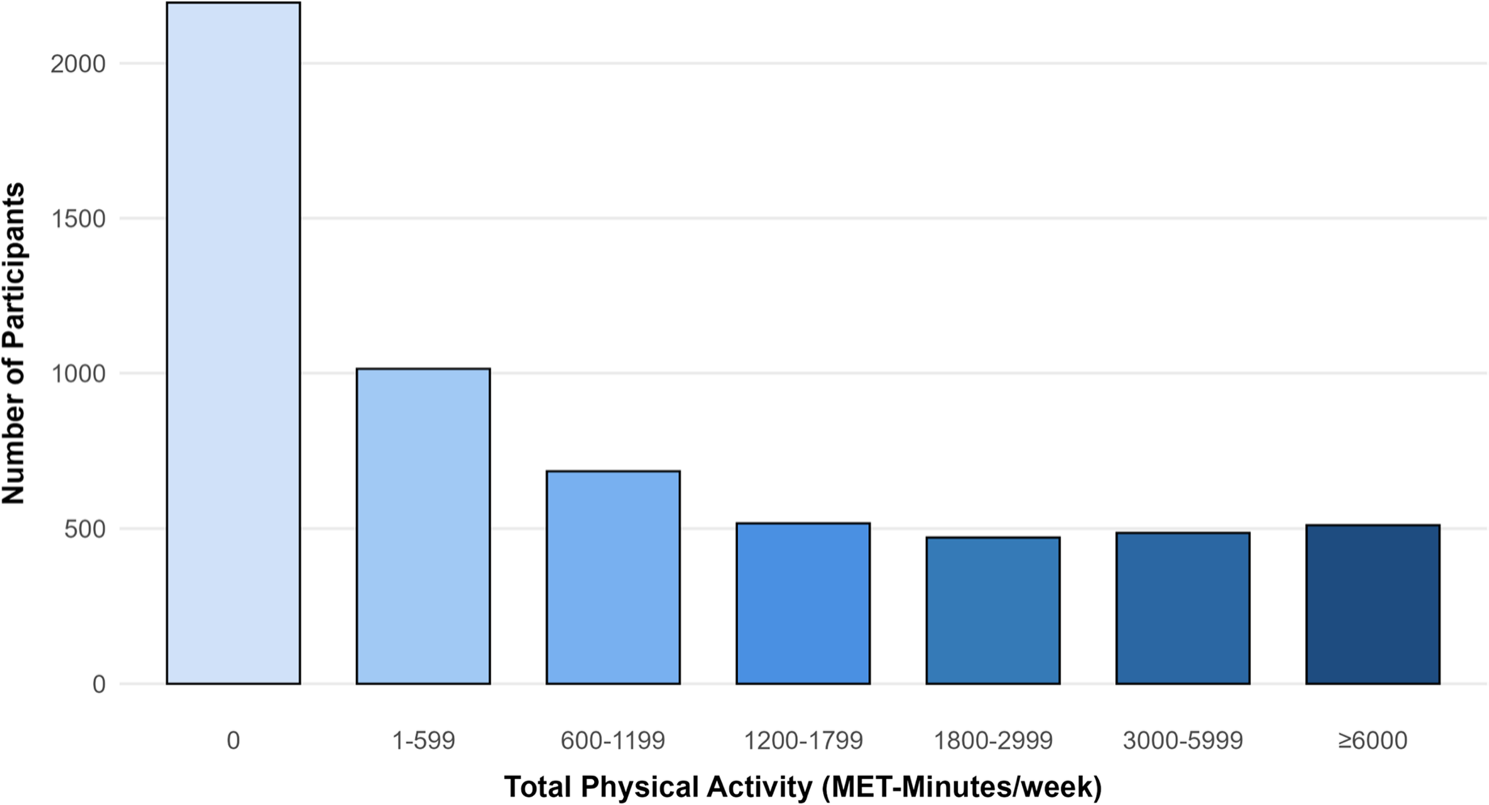
Distribution of total physical activity in menopausal population. Abbreviation: MET: Metabolic equivalent of task

Data of 30,213 female participants were extracted from the database from the period 2007 to 2018. After these exclusions, 5,880 postmenopausal women (median age: 63 years, IQR: 56–71) were included in the final analysis (Fig. 1). The median age at menopause was 47 years (IQR: 40–51). Among the total participants, 76.6% (*n* = 2799) were non-Hispanic White, 27.2% (*n* = 1210) had more than a college education, 59.5% (*n* = 3011) were married or living with partner. The prevalence rates of comorbidities were as follows: 3.9% (*n* = 278) for congestive heart failure, 4.5% (*n* = 276) for coronary heart disease, 5.1% (*n* = 364) for stroke, 15.3% (*n* = 1182) for diabetes, 51.7% (*n* = 3391) for hypertension, 51.8% (*n* = 3129) for hyperlipidemia and 10.1% (*n* = 677) for depression. Overall, 32.0% of participants reported no TPA, 17.2% reported low TPA, and 50.8% achieved high TPA (≥ 600 MET-min/week). The detailed information on the population with varied levels of TPA was summarized in Table 1. The distribution of participants across TPA levels is shown in Fig. 2.

**Table 1:** Baseline characteristics of post-menopausal participants by TPA categories

	Overall	No TPA	Insufficiently active TPA	Sufficiently active TPA	*p* value
Number	5880	2196 (32.0%)	1014 (17.2%)	2670 (50.8%)	
Age, (median, IQR)	63 (56,71)	65 (57–74)	63 (56–71)	62 (55–69)	**< 0.001**
Age at menopause, (median, IQR)	47 (40–51)	46 (40–50)	47 (40–51)	47 (40–51)	**< 0.001**
Race, n (%)					**< 0.001**
Mexican American	694 (4.3)	324 (6.0)	107 (3.6)	263 (3.4)	
Other Hispanic	647 (4.0)	254 (4.6)	92 (3.1)	301 (4.0)	
Non-Hispanic White	2799 (76.6)	987 (73.1)	485 (76.3)	1327 (78.8)	
Non-Hispanic Black	1246 (9.7)	477 (11.0)	237 (11.0)	532 (8.4)	
Other Race	494 (5.4)	154 (5.3)	93 (5.9)	247 (5.3)	
BMI, (median, IQR)	29.2 (25.1–34.2)	30.0 (25.7–35.3)	29.7 (25.4–34.4)	28.3 (24.5–33.1)	**< 0.001**
PIR, n (%)					**< 0.001**
≥ 500%	1062 (28.4)	262 (18.4)	185 (30.8)	615 (33.8)	
≥ 300%	1128 (23.7)	370 (21.9)	213 (22.9)	545 (25.1)	
> 100%	2567 (36.5)	1046 (44.6)	434 (35.4)	1087 (31.7)	
≤ 100%	1123 (11.5)	518 (15.1)	182 (10.8)	423 (9.4)	
Educational level, n (%)					**< 0.001**
Less than 9th grade	612 (4.7)	320 (7.8)	89 (4.0)	203 (3.0)	
9-11th grade	785 (9.6)	369 (13.4)	129 (8.0)	287 (7.8)	
High school graduate/GED or equivalent	1433 (25.3)	568 (28.5)	249 (24.4)	616 (23.6)	
Some college or AA degree	1840 (33.2)	635 (32.4)	333 (35.3)	872 (33.0)	
College graduate or above	1210 (27.2)	304 (18.0)	214 (28.3)	692 (32.6)	
Marital status, n (%)					**< 0.001**
Married/Living with partner	3011 (59.5)	1056 (54.3)	503 (57.7)	1452 (63.4)	
Not married nor living with a partner	2869 (40.5)	1140 (45.7)	511 (42.3)	1218 (36.6)	
Alcohol use, n (%)					**< 0.001**
Yes	3095 (61.6)	1039 (54.5)	548 (63.4)	1508 (65.4)	
No	2785 (38.4)	1157 (45.5)	466 (36.6)	1162 (34.6)	
Smoking status, n (%)					0.471
Yes	2409 (43.5)	922 (44.5)	442 (44.8)	1045 (42.5)	
No	3471 (56.5)	1274 (55.5)	572 (55.2)	1625 (57.5)	
Congestive heart failure, n (%)					**< 0.001**
Yes	278 (3.9)	155 (6.1)	44 (3.4)	79 (2.6)	
No	5602 (96.1)	2041 (93.9)	970 (96.6)	2591 (97.4)	
Coronary heart disease, n (%)					**0.003**
Yes	276 (4.5)	140 (6.1)	45 (4.4)	91 (3.5)	
No	5604 (95.5)	2056 (93.9)	969 (95.6)	2579 (96.5)	
Stroke, n (%)					**< 0.001**
Yes	364 (5.1)	188 (7.7)	55 (4.0)	121 (3.7)	
No	5516 (94.9)	2008 (92.3)	959 (96.0)	2549 (96.3)	
Diabetes, n (%)					**< 0.001**
Yes	1182 (15.3)	578 (22.0)	204 (13.6)	400 (11.6)	
No	4493 (81.4)	1546 (74.4)	777 (82.7)	2170 (85.4)	
Borderline	205 (3.3)	72 (3.6)	33 (3.7)	100 (3.0)	
Hypertension, n (%)					**< 0.001**
Yes	3391 (51.7)	1441 (63.4)	587 (52.2)	1363 (44.2)	
No	2489 (48.3)	755 (36.6)	427 (47.8)	1307 (55.8)	
Hyperlipidemia n (%)					**< 0.001**
Yes	3129 (51.8)	1236 (56.8)	547 (52.1)	1346 (48.6)	
No	2751 (48.2)	960 (43.2)	467 (47.9)	1324 (51.4)	
Depression n (%)					**< 0.001**
Yes	677 (10.1)	318 (14.1)	116 (9.4)	243 (7.7)	
No	5203 (89.9)	1878 (85.9)	898 (90.6)	2427 (92.3)	
MHT n (%)					0.655
Yes	2122 (42.0)	738 (40.8)	397 (42.8)	987 (42.5)	
No	3758 (58.0)	1458 (59.2)	617 (57.2)	1683 (57.5)	
Oophorectomy history					0.134
Yes	1399 (24.6)	562 (26.9)	246 (23.7)	591 (23.5)	
No	4481 (75.4)	1634 (73.1)	768 (76.3)	2079 (76.5)	

*Abbreviations*: *TPA *Total physical activity, *IQR *Interquartile range, *BMI *Body mass index, *PIR *Poverty-to-income ratio, *MHT *Menopausal hormone therapy

### Association between PA and mortality

**Fig. 3:**
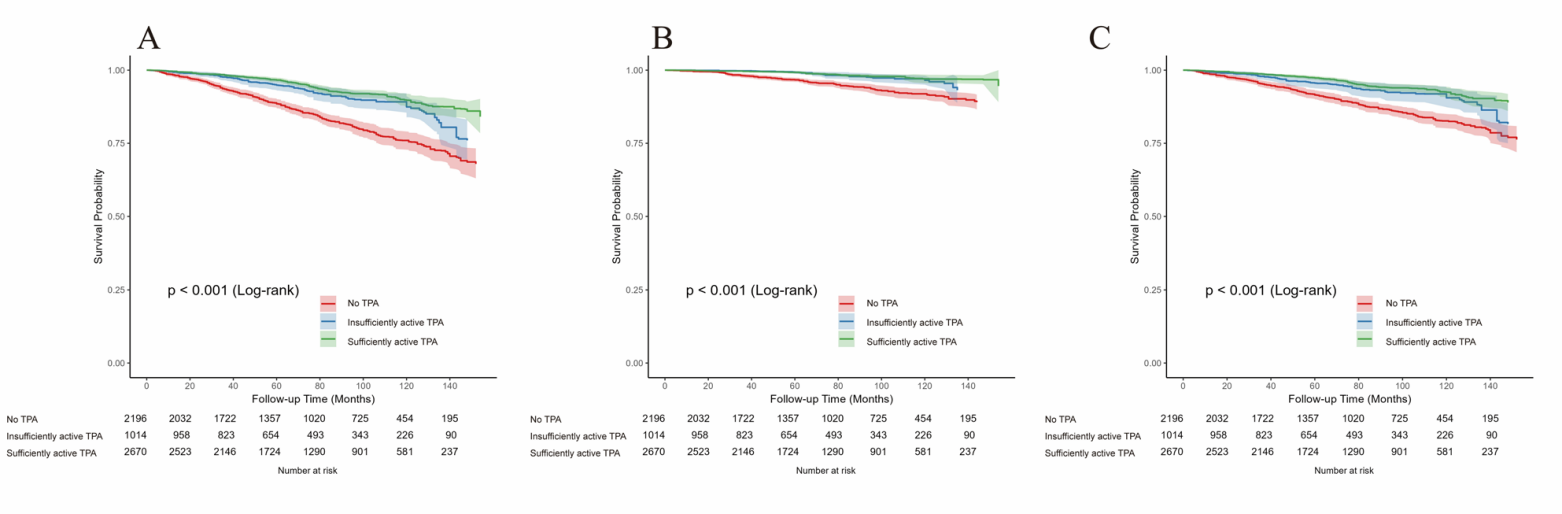
Kaplan–Meier survival curves for all-cause, CVD, and non-CVD mortality by total physical activity levels. (**A**): All-cause mortality, (**B**): CVD mortality, (**C**): non-CVD mortality. All survival curves were weighted using NHANES sampling weights and accounted for complex survey design. Abbreviations: NHANES: National Health and Nutrition Examination Survey; TPA: Total physical activity; CVD: Cardiovascular disease.

During a median follow-up of 77 months, 718 deaths were observed, including 215 CVD deaths and 503 non-CVD deaths. The incidence rates of all-cause mortality in participants with high TPA (9.79/1000 person-years) and low TPA (13.44/1000 person-years) were lower than no TPA participants (26.57/1000 person-years). Kaplan–Meier survival curves showed that participants with higher levels of TPA had higher survival probabilities across all three outcomes (log-rank *p* < 0.001; Fig. 3A and C). Similar patterns were observed for LTPA and OPA, with significantly different survival curves across groups according to log-rank tests (all log-rank *p* < 0.001; Figure S2 and Figure S3).

The association between TPA and mortality is shown in Table 2. Compared with participants with no TPA, both low TPA (unadjusted HR 0.50, 95% CI 0.39–0.66) and high TPA (unadjusted HR 0.36, 95% CI 0.29–0.45) participants were at lower risk of all-cause mortality. After adjustment for potential confounders in Model 3, either low TPA (adjusted HR 0.67, 95% CI 0.53–0.84) or high TPA (adjusted HR 0.63, 95% CI 0.51–0.78) remained significantly associated with a lower risk of all-cause mortality. Looking at CVD mortality, in the fully adjusted Model 3, the adjusted HR of CVD death was 0.60 (95% CI: 0.37–0.97) for low TPA and 0.65 (95% CI: 0.44–0.94) for high TPA, compared with no TPA. In terms of non-CVD mortality, after adjustment for potential confounders, participants with low TPA (adjusted HR 0.69, 95% CI 0.53–0.92) and high TPA (adjusted HR 0.63, 95% CI 0.49–0.80) were at lower risk compared to those with no TPA.

**Table 2:** Association between total physical activity and mortality in post-menopausal participants

Mortality Outcome	Deaths/ Participants	Unadjusted	Model 1	Model 2	Model 3
**All-cause mortality**	**718/5880**	**HR** ** (95% CI)**	**HR (95% CI)**	**HR** **(95% CI)**	**HR** ** (95% CI)**
No TPA	382/2196	[Reference]	[Reference]	[Reference]	[Reference]
Insufficiently active TPA	114/1014	0.50 (0.39–0.66)	0.60 (0.47–0.77)	0.62 (0.48–0.80)	0.67 (0.53–0.84)
Sufficiently active TPA	222/2670	0.36 (0.29–0.45)	0.50 (0.41–0.61)	0.56 (0.46–0.69)	0.63 (0.51–0.78)
**CVD mortality**	**215/5880**	**HR (95% CI)**	**HR (95% CI)**	**HR (95% CI)**	**HR (95% CI)**
No TPA	121/2196	[Reference]	[Reference]	[Reference]	[Reference]
Insufficiently active TPA	32/1014	0.40 (0.24–0.67)	0.51 (0.31–0.86)	0.54 (0.31–0.93)	0.60 (0.37–0.97)
Sufficiently active TPA	62/2670	0.30 (0.20–0.45)	0.45 (0.31–0.67)	0.55 (0.37–0.80)	0.65 (0.44–0.94)
**Non-CVD** **mortality**	**503/5880**	**HR (95% CI)**	**HR (95% CI)**	**HR (95% CI)**	**HR (95% CI)**
No PA	261/2196	[Reference]	[Reference]	[Reference]	[Reference]
Insufficiently active TPA	82/1014	0.55 (0.40–0.74)	0.64 (0.48–0.85)	0.65 (0.49–0.87)	0.69 (0.53–0.92)
Sufficiently active TPA	160/2670	0.39 (0.31–0.50)	0.52 (0.41–0.65)	0.57 (0.45–0.72)	0.63 (0.49–0.80)

Model 1 adjusted for age, age at menopause, and race

Model 2 adjusted for age, race, BMI, PIR, education level, marital status, alcohol consumption, and smoking status

Model 3 adjusted for the variables in Model 2 plus coronary heart disease, congestive heart failure, stroke, diabetes, hypertension, hyperlipidemia, MHT, depression, and oophorectomy history

*Abbreviations*: *TPA *Total physical activity, *HR *Hazard ratio; 95% CI: 95% Confidence interval; *CVD *Cardiovascular disease, *BMI *Body mass index, *PIR *Poverty-to-income ratio, *MHT *Menopausal hormone therapy

In the fully adjusted model, compared with participants with no LTPA, those with low LTPA had a significantly lower risk of all-cause mortality (adjusted HR 0.61, 95% CI: 0.49–0.77) and non-CVD mortality (adjusted HR 0.62, 95% CI: 0.45–0.85), while the association with CVD mortality was not statistically significant (adjusted HR 0.61, 95% CI: 0.37–1.02). Similarly, high LTPA was significantly associated with reduced risks of all-cause (adjusted HR 0.52, 95% CI: 0.39–0.71) and non-CVD mortality (adjusted HR 0.49, 95% CI: 0.34–0.70), but not with CVD mortality (adjusted HR 0.67, 95% CI: 0.42–1.05) (Table S1). Compared with no OPA, low OPA was significantly associated with reduced risks of all-cause mortality (adjusted HR 0.69, 95% CI: 0.50–0.94) and non-CVD mortality (adjusted HR 0.67, 95% CI: 0.47–0.94), whereas the association with CVD mortality was not significant (adjusted HR 0.74, 95% CI: 0.41–1.32). High OPA was not significantly associated with all-cause (adjusted HR 0.79, 95% CI: 0.61–1.03), CVD (adjusted HR 0.78, 95% CI: 0.52–1.19), or non-CVD mortality (adjusted HR 0.80, 95% CI: 0.60–1.06, Table S2).

The associations between domains of PA and cause-specific mortality risk using the Fine–Gray competing risk models are shown in Table S3. High TPA (adjusted HR 0.71, 95% CI: 0.58–0.87), low LTPA (adjusted HR 0.65, 95% CI: 0.49–0.86), and high LTPA (adjusted HR 0.58, 95% CI: 0.45–0.77) were significantly associated with lower non-CVD mortality, whereas low TPA showed only a borderline association (adjusted HR 0.79, 95% CI: 0.61–1.02). No significant associations were observed for CVD mortality with either TPA or LTPA. OPA was not associated with mortality from either CVD or non-CVD.

### Dose–response associations between PA and mortality

**Fig. 4:**
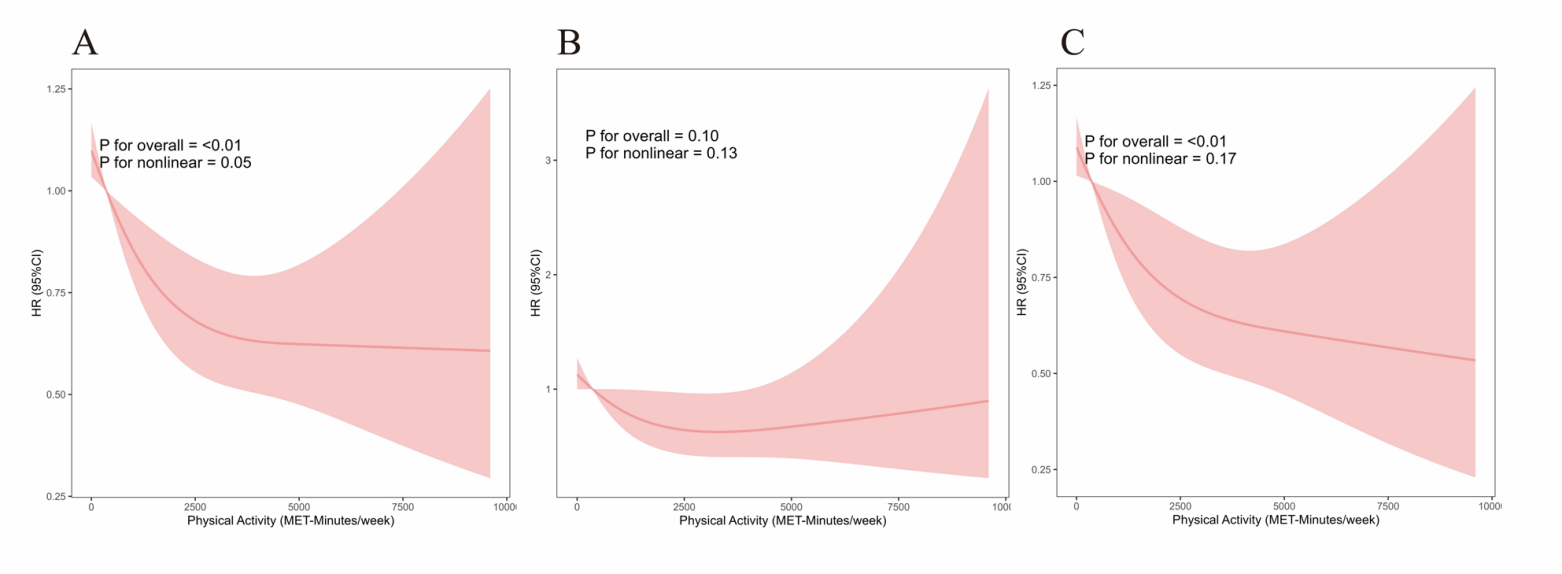
Associations between total physical activity and mortality risk using restricted cubic spline models. (A): All-cause mortality, (B): CVD mortality, (C): non-CVD mortality. All survival curves were weighted using NHANES sampling weights and accounted for complex survey design and adjusted for age, age at menopause, race, BMI, PIR, education level, marital status, alcohol consumption, smoking status, coronary heart disease, congestive heart failure, stroke, diabetes, hypertension, hyperlipidemia, MHT, depression, and oophorectomy history.Abbreviations: NHANES: National Health and Nutrition Examination Survey; HR: Hazard ratio; 95% CI: 95% Confidence interval; MET: Metabolic equivalent of task; CVD: Cardiovascular disease; BMI: Body mass index; PIR: Poverty-to-income ratio; MHT: Menopausal hormone therapy.

RCS analyses were conducted to examine the dose–response associations of TPA, LTPA and OPA with mortality risk. Higher TPA levels were significantly associated with lower risks of all-cause and non-CVD mortality (p for overall < 0.01), with no evidence of nonlinear associations (p for nonlinear > 0.05). We detected no significant association between TPA and CVD mortality (p for overall = 0.10, p for nonlinear = 0.13, Figs. 4).

There was some evidence of a nonlinear association between LTPA and mortality from all causes and non-CVD mortality (p for overall < 0.01; p for nonlinear < 0.01 for all-cause mortality; p for nonlinear = 0.01 for non-CVD mortality), with the steepest risk reductions observed at lower activity levels, followed by a gradual plateau around approximately 2,000 MET-min/week (Figures S4). We detected no significant dose-response association was detected between LTPA and CVD mortality (p for overall = 0.11, p for nonlinear = 0.29, Figure S4). We detected no significant dose-response associations were observed between OPA and any mortality outcomes (all *p* > 0.05, Figures S5).

### Associations of graded PA levels with mortality

Table 3 shows the associations of graded TPA levels with mortality. Compared with the recommended level of 600–1199 MET-min/week, higher TPA levels did not confer consistent additional benefits for all-cause, CVD, or non-CVD mortality. Nonetheless, significant trends across categories were observed for all-cause (p for trend < 0.001) and non-CVD mortality (p for trend = 0.003), but not for CVD mortality (p for trend = 0.059).

**Table 3:** Association of different levels of total physical activity and mortality in full-adjusted models

PA group (MET-min/week)	All-cause mortality		CVD mortality		Non-CVD mortality	
	Deaths/ Participants	HR (95% CI)	Deaths/ Participants	HR (95% CI)	Deaths/ Participants	HR (95% CI)
Total	718/5880		215/5880		503/5880	
0	382/2196	1.77 (1.32–2.37)	121/2196	1.35 (0.80–2.27)	261/2196	1.96 (1.35–2.85)
1-599	114/1014	1.18 (0.83–1.69)	32/1014	0.80 (0.44–1.47)	82/1014	1.36 (0.88–2.11)
600–1199	65/685	[Reference]	20/685	[Reference]	45/685	[Reference]
1200–1799	62/517	1.43 (0.98–2.06)	15/517	0.77 (0.37–1.63)	47/517	1.72 (1.09–2.73)
1800–2999	34/471	1.14 (0.68–1.94)	8/471	0.78 (0.35–1.77)	26/471	1.32 (0.70–2.47)
3000–5999	31/486	1.00 (0.62–1.63)	9/486	0.94 (0.33–2.70)	22/486	1.04 (0.56–1.94)
≥ 6000	30/511	0.98 (0.64–1.52)	10/511	0.71 (0.30–1.68)	20/511	1.10 (0.63–1.93)
p for trend	<0.001		0.059		0.003	

Models were adjusted for age, age at menopause, race, BMI, PIR, education level, marital status, alcohol consumption, smoking status, coronary heart disease, congestive heart failure, stroke, diabetes, hypertension, hyperlipidemia, MHT, depression, and oophorectomy history

*Abbreviations*: *HR *Hazard ratio, 95% *CI*: 95% Confidence interval, *CVD *Cardiovascular disease, *MET *Metabolic equivalent of task, *BMI *Body mass index, *PIR *Poverty-to-income ratio, *MHT *Menopausal hormone therapy

The association of mortality outcomes with LTPA and OPA are shown in Tables S4 and Table S5. Higher levels beyond 600–1199 MET-min/week LTPA did not confer consistent additional benefits, although significant linear trends were detected for all-cause (p for trend < 0.001), CVD (*p* = 0.028), and non-CVD mortality (*p* < 0.001). In contrast, OPA showed no significant linear trends for all-cause (*p* = 0.170), CVD (*p* = 0.348), or non-CVD mortality (*p* = 0.241).

### Sensitivity analyses

A sensitivity analysis excluding deaths within the first 24 months of follow-up yielded consistent findings with the main results (Tables 4 and 5). Significant trends across categories were evident for TPA (p for trend = 0.005 for all-cause, 0.020 for CVD, and 0.027 for non-CVD mortality) and for LTPA (p for trend < 0.001 for all-cause and non-CVD mortality, and 0.006 for CVD mortality). We detected no significant trends across categories for OPA (p for trend > 0.05).

**Table 4:** Associations of total, leisure-time, and occupational physical activity with all-cause, cardiovascular, and non-cardiovascular mortality after excluding deaths within the first 24 months of follow-up

PA categories	All- cause mortality		CVD mortality		Non-CVD mortality	
	**Deaths/ Participants**	**Adjusted model**	**Deaths/ Participants**	**Adjusted model**	**Deaths/ Participants**	**Adjusted model**
**Overall**	**593/5755**		**182/5755**		**411/5755**	
**TPA**						
No TPA	310/2124	[Reference]	102/2124	[Reference]	208/2124	[Reference]
Insufficiently active TPA	99/999	0.69 (0.55–0.88)	30/999	0.63 (0.39–1.03)	69/999	0.71 (0.53–0.97)
Sufficiently active TPA	184/2632	0.62 (0.49–0.79)	50/2632	0.57 (0.38–0.84)	134/2632	0.65 (0.50–0.84)
**LTPA**						
No LTPA	441/3436	[Reference]	137/3436	[Reference]	304/3436	[Reference]
Low LTPA	72/948	0.62 (0.48–0.80)	22/948	0.57 (0.34–0.93)	50/948	0.64 (0.45–0.91)
High LTPA	80/1371	0.51 (0.37–0.71)	23/1371	0.57 (0.36–0.90)	57/1371	0.49 (0.33–0.73)
**OPA**						
No OPA	456/3922	[Reference]	140/3922	[Reference]	316/3922	[Reference]
Low OPA	43/450	0.71 (0.51–0.99)	15/450	0.86 (0.49–1.51)	28/450	0.65 (0.44–0.96)
High OPA	94/1383	0.81 (0.60–1.09)	27/1383	0.73 (0.46–1.16)	67/1383	0.84 (0.61–1.14)

Models were adjusted for age, age at menopause, race, BMI, PIR, education level, marital status, alcohol consumption, smoking status, coronary heart disease, congestive heart failure, stroke, diabetes, hypertension, hyperlipidemia, MHT, depression, and oophorectomy history

*Abbreviations*: *PA *Physical activity, *TPA *Total physical activity, *LTPA *Leisure-time physical activity, *OPA *Occupational physical activity, *HR *Hazard ratio, 95% *CI *95% Confidence interval, *CVD *Cardiovascular disease, *MET *Metabolic equivalent of task, *BMI *Body mass index, *PIR *Poverty-to-income ratio, *MHT *Menopausal hormone therapy

**Table 5:** Associations of different levels of total, leisure-time, and occupational physical activity with all-cause, cardiovascular, and non-cardiovascular mortality after excluding deaths within the first 24 months of follow-up

	All-cause mortality		CVD mortality		Non-CVD mortality	
**TPA group (MET-min/week)**	**Deaths/ Participants**	**HR (95% CI)**	**Deaths/ Participants**	**HR (95% CI)**	**Deaths/ Participants**	**HR (95% CI)**
Total	593/5755		182/5755		411/5755	
0	310/2124	1.92 (1.39–2.66)	102/2124	1.65 (0.89–3.06)	208/2124	2.04 (1.35–3.07)
1-599	99/999	1.33 (0.90–1.96)	30/999	1.04 (0.52–2.09)	69/999	1.46 (0.90–2.36)
600–1199	49/669	[Reference]	15/669	[Reference]	34/669	[Reference]
1200–1799	52/507	1.51 (1.04–2.17)	12/507	0.82 (0.37–1.81)	40/507	1.82 (1.14–2.91)
1800–2999	29/466	1.24 (0.70–2.18)	7/466	0.98 (0.40–2.37)	22/466	1.37 (0.67–2.79)
3000–5999	29/484	1.19 (0.75–1.89)	8/484	1.07 (0.39–2.93)	21/484	1.25 (0.67–2.32)
≥ 6000	25/506	1.34 (0.71–1.84)	8/506	0.71 (0.28–1.83)	17/506	1.31 (0.72–2.39)
p for trend	0.005		0.020		0.027	
**LTPA group (MET-min/week)**	**Deaths/Participants**	**HR (95% CI)**	**Deaths/Participants**	**HR (95% CI)**	**Deaths/Participants**	**HR (95% CI)**
Total	593/5755		182/5755		411/5755	
0	441/3436	2.06 (1.39–3.06)	137/3436	1.50 (0.85–2.64)	304/3436	2.36 (1.42–3.91)
1-599	72/948	1.27 (0.77–2.09)	22/948	0.85 (0.41–1.74)	50/948	1.51 (0.79–2.90)
600–1199	39/638	[Reference]	13/638	[Reference]	26/638	[Reference]
1200–1799	23/341	1.15 (0.65–2.03)	6/341	0.75 (0.25–2.23)	17/341	1.36 (0.71–2.61)
1800–2999	13/241	1.38 (0.72–2.66)	2/241	0.82 (0.18–3.80)	11/241	1.66 (0.78–3.56)
≥ 3000	5/151	0.70 (0.25–1.94)	2/151	0.46 (0.09–2.47)	3/151	0.80 (0.22–2.97)
p for trend	<0.001		0.006		<0.001	
**OPA group (MET-min/week)**	**Deaths/Participants**	**HR (95% CI)**	**Deaths/Participants**	**HR (95% CI)**	**Deaths/Participants**	**HR (95% CI)**
Total	593/5755		182/5755		411/5755	
0	456/3922	2.25 (1.11–4.58)	140/3922	1.43 (0.47–4.34)	316/3922	2.79 (1.26–6.18)
1-599	43/450	[Reference]	15/450	[Reference]	28/450	[Reference]
600–1199	12/246	1.60 (0.72–3.62)	4/246	1.23 (0.38–3.98)	8/246	1.82 (0.71–4.67)
1200–1799	26/238	2.17 (1.02–4.60)	6/238	0.75 (0.17–3.34)	20/238	3.18 (1.32–7.65)
1800–2999	16/229	2.28 (0.90–5.78)	3/229	0.86 (0.17–4.43)	13/229	3.25 (1.18–8.90)
3000–5999	16/286	1.48 (0.64–3.43)	6/286	1.56 (0.51–4.75)	10/286	1.42 (0.46–4.37)
≥ 6000	24/384	2.38 (1.13–5.02)	8/384	1.10 (0.29–4.22)	16/384	3.20 (1.37–7.46)
p for trend	0.336		0.311		0.487	

Models were adjusted for age, age at menopause, race, BMI, PIR, education level, marital status, alcohol consumption, smoking status, coronary heart disease, congestive heart failure, stroke, diabetes, hypertension, hyperlipidemia, MHT, depression, and oophorectomy history

*Abbreviations*: *PA *Physical activity, *TPA *Total physical activity, *LTPA *Leisure-time physical activity, *OPA *Occupational physical activity, *HR *Hazard ratio, 95% *CI *95% Confidence interval, *CVD *Cardiovascular disease, *MET *Metabolic equivalent of task, *BMI *Body mass index, *PIR* Poverty-to-income ratio, *MHT *Menopausal hormone therapy

Tables S6–S7 shows the associations of TPA, LTPA and OPA with mortality outcomes in a sensitivity analysis after multiple imputation for missing covariates. Tests for trend across categories showed significant associations for either TPA (p for trend < 0.001 for all-cause and non-CVD mortality, 0.005 for CVD mortality) or LTPA (p for trend < 0.001 for all-cause and non-CVD mortality, 0.008 for CVD mortality). OPA was significantly associated with all-cause mortality (p for trend = 0.003) and non-CVD mortality (p for trend = 0.007), but not with CVD mortality (p for trend = 0.051).

## Discussion

In this nationally representative cohort of postmenopausal women, higher levels of TPA and LTPA were significantly associated with reduced risks of all-cause and non-CVD mortality. These findings support an association between adequate PA and lower mortality risk among postmenopausal women.

Our findings align with some previous literature on PA and mortality. For example, the Iowa Women’s Health Study reported a graded reduction in mortality risk with increasing exercise frequency among postmenopausal women [13]. Women exercising at least four times per week had roughly a 38% lower mortality risk compared to those rarely or never active. Pooled data of six cohort-studies showed that mortality benefits plateaued at approximately 3–5 times the minimum recommended LTPA level, with only modest additional gains beyond that point and no evidence of harm at very high activity doses [6]. This is in line with our observation that beyond roughly 2,000 MET-min/week of LTPA, no further mortality reduction was evident. When comparing higher exposure categories (≥ 1200 MET-min/week) directly with the reference group (600–1199 MET-min/week), no consistent additional benefits were observed. However, the consistently significant linear trends for LTPA indicate that women engaging in activity at higher levels may still experience lower mortality risks. Notably, while categorical Cox models indicated inverse associations with CVD mortality, these were not confirmed in the RCS analyses, which showed no significant overall or nonlinear associations. Given the relatively limited number of cardiovascular deaths, the lack of statistically significant associations for cardiovascular mortality likely reflects limited statistical power rather than a true absence of association.

While LTPA is clearly linked to better health outcomes, several studies have shown that high OPA is associated with increased mortality, a phenomenon known as the physical activity paradox. The Copenhagen General Population Study showed that LTPA was associated with reduced CVD and all-cause mortality risk, while higher OPA was associated with higher risks [14]. A pooled analysis of 23 studies with 655 892 participants showed that OPA was not associated with reduced CVD mortality (HR for males: 1.00 [0.87–1.15], HR for females: 0.95 [0.82–1.09]) [26]. Our analyses showed no significant association between OPA and mortality outcomes, which is in line with the above-mentioned studies. Our spline analysis showed a steep reduction in mortality risk from 0 to around 600–1200 MET-min/ week, followed by a plateau and then an apparent increase at very high levels of total PA. This non-linear pattern is consistent with previous evidence [27, 28] that the most significant relative gains come from moving out of inactivity, while additional activity confers smaller incremental benefits. The apparent flattening of the curve at > 4000 MET-min/ week may reflect inclusion of occupational PA in the total score, a phenomenon known as the “physical activity paradox”. The apparent plateau could reflect the inclusion of work-related activity, not the absence of biological benefit from higher leisure PA. This may provide a plausible explanation for why higher levels of total PA did not show additional benefits. Notably, OPA as measured in NHANES does not capture important dimensions such as task variability, biomechanical load, recovery opportunities, or psychosocial stressors. Thus, the null or non-protective associations observed for OPA could be influenced by limitations in exposure characterization, rather than reflecting an inherent lack of benefit of occupational activity per se.

The biological plausibility of our findings is supported by several well-established physiological pathways. PA has been shown to enhance cardiovascular function through improving endothelial function, lipid profiles, blood pressure regulation, and insulin sensitivity [29–31]. It also plays a crucial role in reducing systemic inflammation, oxidative stress, and autonomic dysfunction [32, 33]—all of which are implicated in the pathogenesis of chronic diseases and premature mortality. In postmenopausal women, the sharp decline in estrogen levels contributes to increased risks of central adiposity, dyslipidemia, insulin resistance, and vascular dysfunction [34, 35]. Adequate PA may help counteract these adverse changes by modulating metabolic pathways, preserving vascular integrity, and attenuating pro-inflammatory processes [36, 37]. These mechanisms collectively provide biological support for the observed associations between higher PA levels and lower mortality risk in this vulnerable population. It is worth noting that these mechanisms are most likely to reflect the benefits of leisure-time and structured exercise, whereas heavy occupational PA may not provide the same physiological benefits.

Given the aging population and the large proportion of women entering menopause each year, our findings are informative for public health decision-making. The greatest relative benefits were achieved when moving from no PA to modest volumes, while the flattening of the dose–response curve at higher levels may be influenced by occupational PA. These results support public health efforts to reduce inactivity and promote PA for healthy aging among postmenopausal women. Importantly, they underscore the clinical and public health relevance of achievable PA thresholds, particularly for counselling postmenopausal women who are currently inactive.

This study has several strengths. First, our analyses leveraged data from a large, nationally representative sample of postmenopausal women with survey weighting, which enhances the generalizability of the findings. Second, long-term mortality follow-up through linkage with the National Death Index may strengthen the robustness of outcome ascertainment. Third, our study has the ability to compare all -cause, CVD, and non-CVD mortality simultaneously. Fourth, we employed restricted cubic spline models to capture potential non-linear dose–response patterns. Several limitations should also be acknowledged. First, PA was self-reported, which may be subject to recall bias and misclassification. Second, PA was assessed at a single time point, which may not fully capture long-term activity patterns or changes over time. Third, women with higher PA may be healthier at baseline, while those with No PA may be less healthy, which may lead to overestimation of protective associations. The hazard ratios for all-cause, CVD, and non-CVD mortality associated with TPA are very similar, suggesting that residual confounding may partly explain the consistent results across all outcomes if women with poorer health status were disproportionately clustered in the no-PA group. Despite adjustment for sociodemographic, lifestyle, and clinical variables, unmeasured factors such as subclinical disease or medication use may still confound the associations. Fourth, as an observational study, causal relationships cannot be established, and the possibility of reverse causation cannot be completely excluded. Fifth, we did not distinguish between natural and surgical menopause, and such differences may confer heterogeneous health risks and potentially influence the observed associations. Notably, the lack of differentiation between natural and surgical menopause may reflect current evidence gaps rather than established equivalence. Although the directed acyclic graph appropriately informed covariate adjustment, it does not resolve questions of biological heterogeneity related to menopause type. Sixth, the postmenopausal population included in this study spanned a wide age range, and how age-related heterogeneity may influence exposure–outcome relationship needs to be investigated in future studies. Finally, the PA categories applied in this study (600–1199, 1200–1799, 1800–2999 or more MET-min/week) were originally developed for LPA [6], which may limit direct comparability with previous studies.

## Conclusions

This cohort study of a nationally representative sample of postmenopausal women found associations between higher levels of PA and lower mortality risks. No additional benefit was detected in this dataset when total PA exceeded guideline level, partly because domain-specific effects differ. These findings reinforce the public health importance of promoting sufficient PA among postmenopausal women to support healthy aging.

## Supplementary Information

Supplementary Material 1.

## Data Availability

The datasets generated and analyzed in the current study are available at NHANES website: https://www.cdc.gov/nchs/nhanes/index.html.

## Abbreviations

PA: Physical activity
TPA: Total physical activity
LTPA: Leisure-time physical activity
OPA: Occupational physical activity
MET: Metabolic equivalent of task
CVD: Cardiovascular disease
NHANES: National Health and Nutrition Examination Survey
NCHS: National Center for Health Statistics
CDC: Centers for Disease Control and Prevention
GPAQ: Global Physical Activity Questionnaire
BMI: Body mass index
PIR: Poverty-to-income ratio
CI: Confidence intervals
RCS: Restricted cubic spline
MHT: Menopausal hormone therapy
