# Supplementary material for: Association between physical activity and mortality in postmenopausal women: evidence from NHANES 2007–2018
Source: BMC Womens Health. 2026 Feb 9;26:160. doi: 10.1186/s12905-026-04315-3 (PMC12983851; doi:10.1186/s12905-026-04315-3)
Supplement: Supplementary file 1 — Supplementary Material 1. [file 12905_2026_4315_MOESM1_ESM.docx]

**Supplemental Material**

Table S1. Association between leisure-time physical activity and mortality in post-menopausal participants.

Table S2. Association between occupational physical activity and mortality in post-menopausal participants.

Table S3. Associations of total, leisure-time, and occupational physical activity with cause-specific mortality in postmenopausal participants (Fine–Gray models).

Table S4. Association of different levels of leisure-time physical activity and mortality in full-adjusted models.

Table S5. Association of different levels of occupational physical activity and mortality in full-adjusted models.

Table S6. Associations of physical activity domains with mortality outcomes after multiple imputation for missing covariates.

Table S7. Associations of different levels of total, leisure-time, and occupational physical activity with mortality outcomes after multiple imputation for missing covariates.

Figure S1. Directed acyclic graph illustrating the assumed causal relationships between physical activity, covariates, and mortality.

Figure S2. Kaplan–Meier survival curves for all-cause, CVD, and non-CVD mortality by leisure time physical activity levels and quartiles.

Figure S3. Kaplan–Meier survival curves for all-cause, CVD, and non-CVD mortality by occupational physical activity levels and quartiles.

Figure S4. Associations between leisure-time physical activity and mortality risk using restricted cubic spline models.

Figure S5. Associations between occupational physical activity and mortality risk using restricted cubic spline models.

**Table S1. Association between leisure-time physical activity and mortality in post-menopausal participants.**

| **Mortality Outcome** | **Deaths/ Participants** | **Unadjusted** | **Model 1** | **Model 2** | **Model 3** |
| --- | --- | --- | --- | --- | --- |
| **All-cause mortality** | **718/5880** | **HR (95% CI)** | **HR (95% CI)** | **HR (95% CI)** | **HR (95% CI)** |
| No LTPA | 539/3534 | [Reference] | [Reference] | [Reference] | [Reference] |
| Low LTPA | 84/960 | 0.48 (0.37-0.64) | 0.55 (0.42-0.71) | 0.60 (0.46-0.78) | 0.61 (0.49-0.77) |
| High LTPA | 95/1386 | 0.34 (0.24-0.46) | 0.42 (0.32-0.55) | 0.49 (0.36-0.66) | 0.52 (0.39-0.71) |
|  | | | | | |
| **CVD mortality** | **215/5880** | **HR (95% CI)** | **HR (95% CI)** | **HR (95% CI)** | **HR (95% CI)** |
| No LTPA | 162/3534 | [Reference] | [Reference] | [Reference] | [Reference] |
| Low LTPA | 25/960 | 0.43 (0.26-0.72) | 0.51 (0.31-0.86) | 0.59 (0.35-0.99) | 0.61 (0.37-1.02) |
| High LTPA | 28/1836 | 0.33 (0.21-0.50) | 0.45 (0.29-0.69) | 0.59 (0.37-0.95) | 0.67 (0.42-1.05) |
|  | | | | | |
| **Non-CVD**  **mortality** | **503/5880** | **HR (95% CI)** | **HR (95% CI)** | **HR (95% CI)** | **HR (95% CI)** |
| No LTPA | 377/3534 | [Reference] | [Reference] | [Reference] | [Reference] |
| Low LTPA | 59/960 | 0.50 (0.35-0.73) | 0.56 (0.40-0.79) | 0.60 (0.43-0.85) | 0.62 (0.45-0.85) |
| High LTPA | 67/1836 | 0.34 (0.23-0.49) | 0.41 (0.30-0.57) | 0.46 (0.32-0.65) | 0.49 (0.34-0.70) |

Model 1 adjusted for age, age at menopause and race.

Model 2 adjusted for age, race, BMI, PIR, education levels, marital, alcohol consumption and smoking status.

Model 3 adjusted for the variables in model 2 plus coronary heart disease, congestive heart failure, stroke, diabetes, hypertension, hyperlipidemia, **MHT, depression and** oophorectomy history.

Abbreviations: LTPA: Leisure-time Physical Activity, HR: Hazard Ratio, 95% CI: 95% Confidence Interval, CVD: Cardiovascular disease, BMI: Body Mass Index, PIR: Poverty Income Ratio, MHT: menopausal hormone therapy.

**Table S2. Association between occupational physical activity and mortality in post-menopausal participants.**

| **Mortality Outcome** | **Deaths/ Participants** | **Unadjusted** | **Model 1** | **Model 2** | **Model 3** |
| --- | --- | --- | --- | --- | --- |
| **All-cause mortality** | **718/5880** | **HR (95% CI)** | **HR (95% CI)** | **HR (95% CI)** | **HR (95% CI)** |
| No OPA | 555/4021 | [Reference] | [Reference] | [Reference] | [Reference] |
| Low OPA | 50/457 | 0.68 (0.51-0.93) | 0.63 (0.47-0.86) | 0.63 (0.45-0.87) | 0.69 (0.50-0.94) |
| High OPA | 113/1402 | 0.51 (0.39-0.68) | 0.69 (0.53-0.90) | 0.74 (0.58-0.96) | 0.79 (0.61-1.03) |
|  | | | | | |
| **CVD mortality** | **215/5880** | **HR (95% CI)** | **HR (95% CI)** | **HR (95% CI)** | **HR (95% CI)** |
| No OPA | 167/4021 | [Reference] | [Reference] | [Reference] | [Reference] |
| Low OPA | 15/457 | 0.72 (0.41-1.24) | 0.69 (0.40-1.17) | 0.65 (0.37-1.14) | 0.74 (0.41-1.32) |
| High OPA | 33/1402 | 0.43 (0.26-0.71) | 0.65 (0.41-1.03) | 0.71 (0.45-1.10) | 0.78 (0.52-1.19) |
|  | | | | | |
| **Non-CVD**  **mortality** | **503/5880** | **HR (95% CI)** | **HR (95% CI)** | **HR (95% CI)** | **HR (95% CI)** |
| No OPA | 388/4021 | [Reference] | [Reference] | [Reference] | [Reference] |
| Low OPA | 35/457 | 0.67 (0.47-0.96) | 0.62 (0.43-0.89) | 0.62 (0.43-0.89) | 0.67 (0.47-0.94) |
| High OPA | 80/1402 | 0.55 (0.41-0.72) | 0.71 (0.54-0.94) | 0.76 (0.58-0.99) | 0.80 (0.60-1.06) |

Model 1 adjusted for age, age at menopause, and race.
Model 2 adjusted for age, race, BMI, PIR, education level, marital status, alcohol consumption, and smoking status.
Model 3 adjusted for the variables in Model 2 plus coronary heart disease, congestive heart failure, stroke, diabetes, hypertension, hyperlipidemia, MHT, depression, and oophorectomy history.
Abbreviations: OPA: Occupational physical activity; HR: Hazard ratio; 95% CI: 95% Confidence interval; CVD: Cardiovascular disease; BMI: Body mass index; PIR: Poverty-to-income ratio; MHT: Menopausal hormone therapy.

**Table S3. Associations of total, leisure-time, and occupational physical activity with cause-specific mortality in postmenopausal participants (Fine–Gray models).**

| **Physical Activity** | **CVD-Mortality** | | **Non-CVD mortality** | |
| --- | --- | --- | --- | --- |
| **Total PA** | **HR (95% CI)** | **P value** | **HR (95% CI)** | **P value** |
| No PA | [Reference] |  | [Reference] |  |
| Insufficiently active PA | 0.78 (0.53-1.16) | 0.224 | 0.79 (0.61-1.02) | 0.067 |
| Sufficiently active PA | 0.8 (0.58-1.11) | 0.187 | 0.71 (0.58-0.87) | 0.001 |
| **Leisure-time PA** |  |  |  |  |
| No PA | [Reference] |  | [Reference] |  |
| Low PA | 0.79 (0.51-1.21) | 0.275 | 0.65 (0.49-0.86) | 0.003 |
| High PA | 0.82 (0.53-1.27) | 0.373 | 0.58 (0.45-0.77) | <0.001 |
| **Occupational PA** |  |  |  |  |
| No PA | [Reference] |  | [Reference] |  |
| Low PA | 0.83 (0.48-1.45) | 0.512 | 0.81 (0.57-1.15) | 0.245 |
| High PA | 0.96 (0.66-1.42) | 0.853 | 0.81 (0.63-1.04) | 0.097 |

Models were adjusted for age, age at menopause, race, BMI, PIR, education level, marital status, alcohol consumption, smoking status, coronary heart disease, congestive heart failure, stroke, diabetes, hypertension, hyperlipidemia, MHT, depression, and oophorectomy history.

Abbreviations: PA: Physical activity; HR: Hazard ratio; 95% CI: 95% Confidence interval; CVD: Cardiovascular disease; BMI: Body mass index; PIR: Poverty-to-income ratio; MHT: Menopausal hormone therapy.

**Table S4. Association of different levels of leisure-time physical activity and mortality in full-adjusted models.**

|  | **All-cause mortality** | | **CVD mortality** | | **Non-CVD mortality** | |
| --- | --- | --- | --- | --- | --- | --- |
| **PA group (MET-min/week)** | **Deaths/ Participants** | **HR (95% CI)** | **Deaths/ Participants** | **HR (95% CI)** | **Deaths/ Participants** | **HR (95% CI)** |
| Total | 718/5880 |  | 215/5880 |  | 503/5880 |  |
| 0 | 539/3534 | 1.73 (1.22-2.46) | 162/3534 | 1.20 (0.68-2.12) | 377/3534 | 1.99 (1.27-3.10) |
| 1-599 | 84/960 | 1.06 (0.70-1.61) | 25/960 | 0.73 (0.36-1.48) | 59/960 | 1.22(0.70-2.13) |
| 600-1199 | 52/651 | [Reference] | 17/651 | [Reference] | 35/651 | [Reference] |
| 1200-1799 | 24/342 | 0.87 (0.50-1.49) | 6/342 | 0.56 (0.18-1.73) | 18/342 | 1.01 (0.54-1.89) |
| 1800-2999 | 13/241 | 0.94 (0.49-1.79) | 2/241 | 0.61 (0.13-2.77) | 11/241 | 1.08 (0.51-2.28) |
| ≥3000 | 6/152 | 0.59 (0.24-1.45) | 3/152 | 0.65 (0.18-2.35) | 3/152 | 0.55 (0.15-1.99) |
| p for trend | ＜0.001 | | 0.028 | | ＜0.001 | |

Models were adjusted for age, age at menopause, race, BMI, PIR, education level, marital status, alcohol consumption, smoking status, coronary heart disease, congestive heart failure, stroke, diabetes, hypertension, hyperlipidemia, MHT, depression, and oophorectomy history.

Abbreviations: PA: Physical activity; TPA: Total physical activity; LTPA: Leisure-time physical activity; OPA: Occupational physical activity; HR: Hazard ratio; 95% CI: 95% Confidence interval; CVD: Cardiovascular disease; MET: Metabolic equivalent of task; BMI: Body mass index; PIR: Poverty-to-income ratio; MHT: Menopausal hormone therapy.

**Table S5. Association of different levels of occupational physical activity and mortality in full-adjusted models.**

|  | **All-cause mortality** | | **CVD mortality** | | **Non-CVD mortality** | |
| --- | --- | --- | --- | --- | --- | --- |
| **PA group (MET-min/week)** | **Deaths/ Participants** | **HR (95% CI)** | **Deaths/ Participants** | **HR (95% CI)** | **Deaths/ Participants** | **HR (95% CI)** |
| Total | 718/5880 |  | 215/5880 |  | 503/5880 |  |
| 0 | 555/4021 | 1.85 (1.09-3.14) | 167/4021 | 1.08 (0.49-2.37) | 388/4021 | 2.34 (1.24-4.42) |
| 1-599 | 50/457 | 1.27 (0.68-2.36) | 15/457 | 0.79 (0.28-2.22) | 35/457 | 1.56 (0.77-3.15) |
| 600-1199 | 19/253 | [Reference] | 6/253 | [Reference] | 13/253 | [Reference] |
| 1200-1799 | 29/241 | 1.62 (0.83-3.17) | 7/241 | 0.53 (0.17-1.70) | 22/241 | 2.38 (1.09-5.22) |
| 1800-2999 | 19/232 | 1.96 (0.95-4.06) | 4/232 | 0.74 (0.20-2.76) | 15/232 | 2.75 (1.23-6.17) |
| 3000-5999 | 18/288 | 1.17 (0.56-2.41) | 7/288 | 1.20 (0.44-3.32) | 11/288 | 1.13 (0.45-2.87) |
| ≥6000 | 28/388 | 1.75 (0.96-3.20) | 9/388 | 0.80 (0.24-2.61) | 19/388 | 2.33 (1.19-4.57) |
| p for trend | 0.170 | | 0.348 | | 0.241 | |

Models were adjusted for age, age at menopause, race, BMI, PIR, education level, marital status, alcohol consumption, smoking status, coronary heart disease, congestive heart failure, stroke, diabetes, hypertension, hyperlipidemia, MHT, depression, and oophorectomy history.

Abbreviations: PA: Physical activity; TPA: Total physical activity; LTPA: Leisure-time physical activity; OPA: Occupational physical activity; HR: Hazard ratio; 95% CI: 95% Confidence interval; CVD: Cardiovascular disease; MET: Metabolic equivalent of task; BMI: Body mass index; PIR: Poverty-to-income ratio; MHT: Menopausal hormone therapy.

**Table S6. Associations of physical activity domains with mortality outcomes after multiple imputation for missing covariates.**

| **PA categories** | **All- cause mortality** | | **CVD mortality** | | **Non-CVD mortality** | |
| --- | --- | --- | --- | --- | --- | --- |
|  | **Deaths/ Participants** | **Adjusted model** | **Deaths/ Participants** | **Adjusted model** | **Deaths/ Participants** | **Adjusted model** |
| **Overall** | 1120/7770 |  | 334/7770 |  | 786/7780 |  |
| **TPA** |  |  |  |  |  |  |
| No TPA | 672/3102 | [Reference] | 215/3102 | [Reference] | 457/3102 | [Reference] |
| Insufficiently active TPA | 159/1285 | 0.63 (0.51-0.78) | 42/1285 | 0.58 (0.36-0.92) | 117/1285 | 0.65 (0.51-0.83) |
| Sufficiently active TPA | 289/3383 | 0.52 (0.43-0.63) | 77/3383 | 0.50 (0.36-0.72) | 212/3383 | 0.53 (0.43-0.66) |
| **LTPA** |  |  |  |  |  |  |
| No LTPA | 888/4903 | [Reference] | 271/4903 | [Reference] | 617/4903 | [Reference] |
| Low LTPA | 115/1182 | 0.59 (0.48-0.74) | 31/1182 | 0.58 (0.38-0.89) | 84/1182 | 0.60 (0.44-0.81) |
| High LTPA | 117/1685 | 0.46 (0.35-0.61) | 32/1685 | 0.54 (0.34-0.84) | 85/1685 | 0.43 (0.31-0.60) |
| **OPA** |  |  |  |  |  |  |
| No OPA | 909/5431 | [Reference] | 275/5431 | [Reference] | 634/5431 | [Reference] |
| Low OPA | 60/559 | 0.65 (0.48-0.87) | 16/559 | 0.60 (0.34-1.07) | 44/559 | 0.67 (0.47-0.95) |
| High OPA | 151/1780 | 0.69 (0.55-0.86) | 43/1780 | 0.64 (0.43-0.96) | 108/1780 | 0.70 (0.55-0.89) |

Models were adjusted for age, age at menopause, race, BMI, PIR, education level, marital status, alcohol consumption, smoking status, coronary heart disease, congestive heart failure, stroke, diabetes, hypertension, hyperlipidemia, MHT, depression, and oophorectomy history.

Abbreviations: PA: Physical activity; TPA: Total physical activity; LTPA: Leisure-time physical activity; OPA: Occupational physical activity; HR: Hazard ratio; 95% CI: 95% Confidence interval; CVD: Cardiovascular disease; MET: Metabolic equivalent of task; BMI: Body mass index; PIR: Poverty-to-income ratio; MHT: Menopausal hormone therapy.

**Table S7. Associations of different levels of total, leisure-time, and occupational physical activity with mortality outcomes after multiple imputation for missing covariates.**

|  | **All-cause mortality** | | **CVD mortality** | | **Non-CVD mortality** | |
| --- | --- | --- | --- | --- | --- | --- |
| **TPA group (MET-min/week)** | **Death/n** | **HR (95% CI)** | **Death/n** | **HR (95% CI)** | **Death/n** | **HR (95% CI)** |
| Total | 1120/7770 |  | 334/7770 |  | 786/7780 |  |
| 0 | 672/3102 | 2.15 (1.63-2.82) | 215/3102 | 1.96 (1.21-3.15) | 457/3102 | 2.20 (1.56-3.10) |
| 1-599 | 159/1285 | 1.35 (0.99-1.86) | 42/1285 | 1.13 (0.65-1.98) | 117/1285 | 1.43 (0.98-2.09) |
| 600-1199 | 82/864 | [Reference] | 22/864 | [Reference] | 60/864 | [Reference] |
| 1200-1799 | 79/647 | 1.46 (1.03-2.08) | 18/647 | 0.88 (0.43-1.80) | 61/647 | 1.68 (1.10-2.54) |
| 1800-2999 | 45/598 | 1.15 (0.72-1.83) | 12/598 | 1.05 (0.54-2.04) | 33/598 | 1.18 (0.67-2.09) |
| 3000-5999 | 45/624 | 0.97 (0.67-1.42) | 13/624 | 1.14 (0.51-2.56) | 32/624 | 0.91 (0.55-1.52) |
| ≥6000 | 38/650 | 0.97 (0.67-1.40) | 12/650 | 0.85 (0.39-1.85) | 26/650 | 1.00 (0.63-1.58) |
| p for trend | <0.001 | | 0.005 | | <0.001 | |
| **LTPA group (MET-min/week)** | **Death/n** | **HR (95% CI)** | **Death/n** | **HR (95% CI)** | **Death/n** | **HR (95% CI)** |
| Total | 1120/7770 |  | 334/7770 |  | 786/7780 |  |
| 0 | 888/4903 | 2.15 (1.53-3.00) | 271/4903 | 1.72 (0.99-2.99) | 617/4903 | 2.32 (1.54-3.49) |
| 1-599 | 115/1182 | 1.27 (0.86-1.87) | 31/1182 | 0.99 (0.51-1.92) | 84/1182 | 1.38 (0.84-2.27) |
| 600-1199 | 60/797 | [Reference] | 17/797 | [Reference] | 43/797 | [Reference] |
| 1200-1799 | 29/417 | 0.93 (0.56-1.53) | 8/417 | 0.73 (0.27-1.95) | 21/417 | 1.01 (0.55-1.83) |
| 1800-2999 | 19/288 | 1.20 (0.70-2.05) | 4/288 | 1.21 (0.40-3.68) | 15/288 | 1.21 (0.63-2.31) |
| ≥3000 | 9/183 | 0.73 (0.36-1.46) | 3/183 | 0.71 (0.19-2.63) | 6/183 | 0.74 (0.30-1.82) |
| p for trend | <0.001 | | 0.008 | | <0.001 | |
| **OPA group (MET-min/week)** | **Death/n** | **HR (95% CI)** | **Death/n** | **HR (95% CI)** | **Death/n** | **HR (95% CI)** |
| Total | 1120/7770 |  | 334/7770 |  | 786/7780 |  |
| 0 | 909/5431 | 1.85 (1.28-2.66) | 275/5431 | 1.56 (0.64-3.83) | 634/5431 | 1.94 (1.28-2.94) |
| 1-599 | 60/559 | [Reference] | 16/559 | [Reference] | 44/559 | [Reference] |
| 600-1199 | 27/308 | 1.20 (0.76-1.89) | 7/308 | 0.94 (0.31-2.88) | 20/308 | 1.30 (0.79-2.13) |
| 1200-1799 | 39/302 | 1.46 (0.89-2.40) | 11/302 | 0.88 (0.30-2.53) | 28/302 | 1.70 (0.95-3.04) |
| 1800-2999 | 24/303 | 1.66 (0.93-2.99) | 5/303 | 0.82 (0.22-3.04) | 19/303 | 1.97 (1.04-3.77) |
| 3000-5999 | 26/370 | 0.94 (0.56-1.57) | 10/370 | 1.32 (0.48-3.61) | 16/370 | 0.79 (0.41-1.51) |
| ≥6000 | 35/497 | 1.43 (0.93-2.18) | 10/497 | 0.96 (0.28-3.31) | 25/497 | 1.58 (0.96-2.60) |
| p for trend | 0.003 | | 0.051 | | 0.007 | |

Models were adjusted for age, age at menopause, race, BMI, PIR, education level, marital status, alcohol consumption, smoking status, coronary heart disease, congestive heart failure, stroke, diabetes, hypertension, hyperlipidemia, MHT, depression, and oophorectomy history.

Abbreviations: PA: Physical activity; TPA: Total physical activity; LTPA: Leisure-time physical activity; OPA: Occupational physical activity; HR: Hazard ratio; 95% CI: 95% Confidence interval; CVD: Cardiovascular disease; MET: Metabolic equivalent of task; BMI: Body mass index; PIR: Poverty-to-income ratio; MHT: Menopausal hormone therapy.


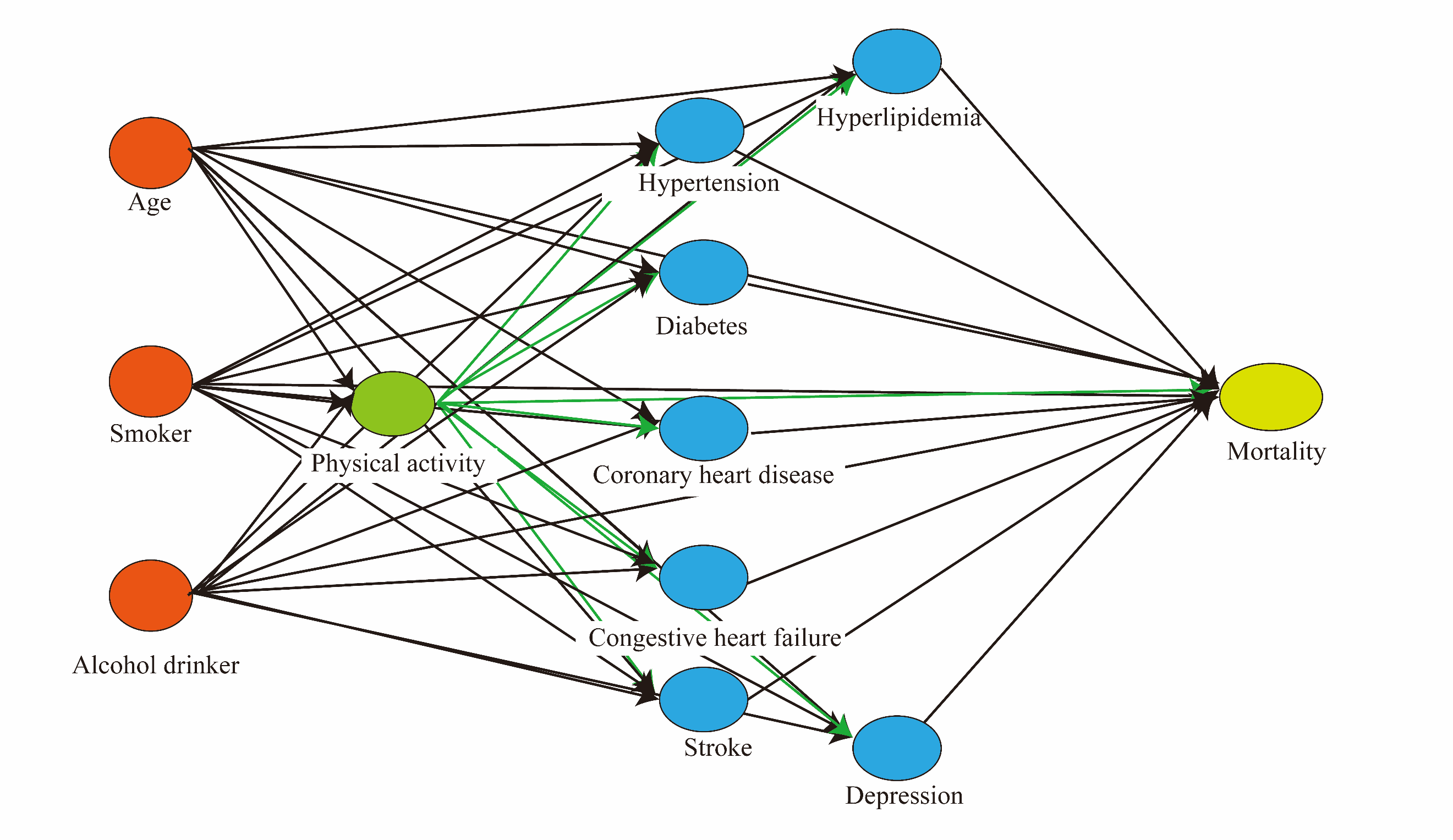


**Figure S1. Directed acyclic graph illustrating the assumed causal relationships between physical activity, covariates, and mortality**


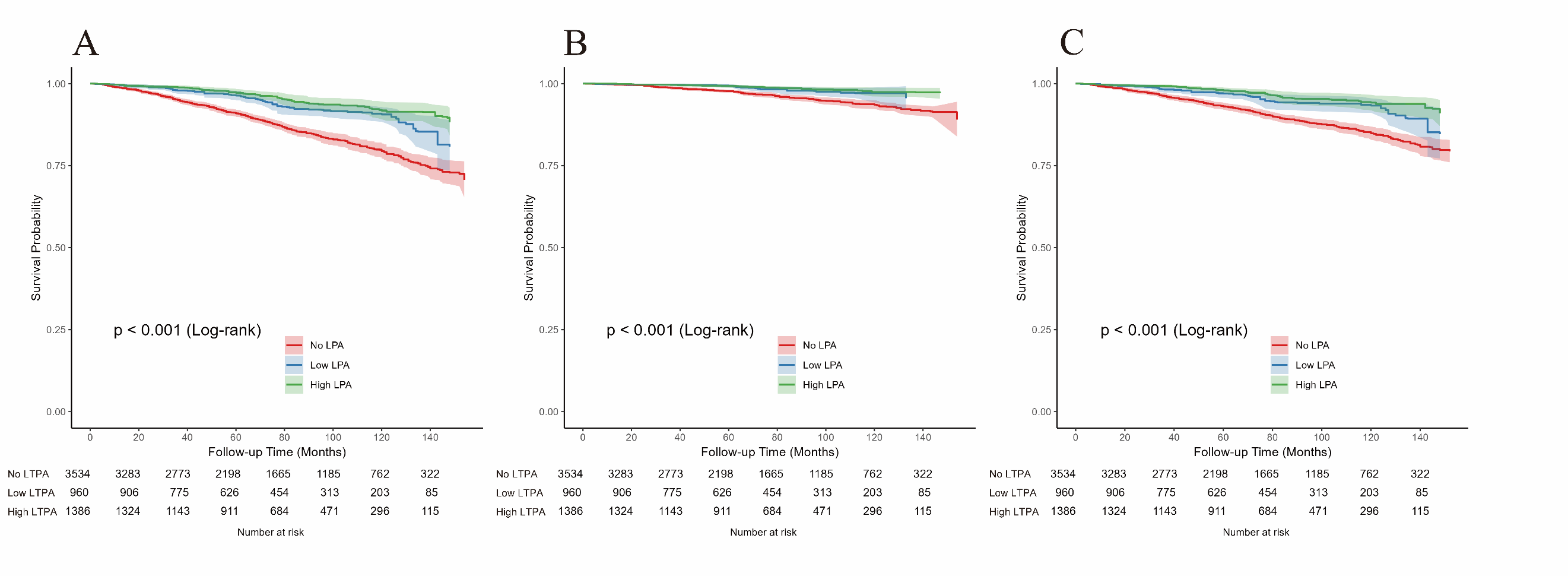


**Figure S2 Kaplan–Meier survival curves for all-cause, CVD, and non-CVD mortality by leisure time physical activity levels and quartiles.**

(A): All-cause mortality, (B): CVD mortality, (C): non-CVD mortality.

All survival curves were weighted using NHANES sampling weights and accounted for complex survey design.

Abbreviations: NHANES: National Health and Nutrition Examination Survey; LTPA: Leisure-time physical activity; CVD: Cardiovascular disease.


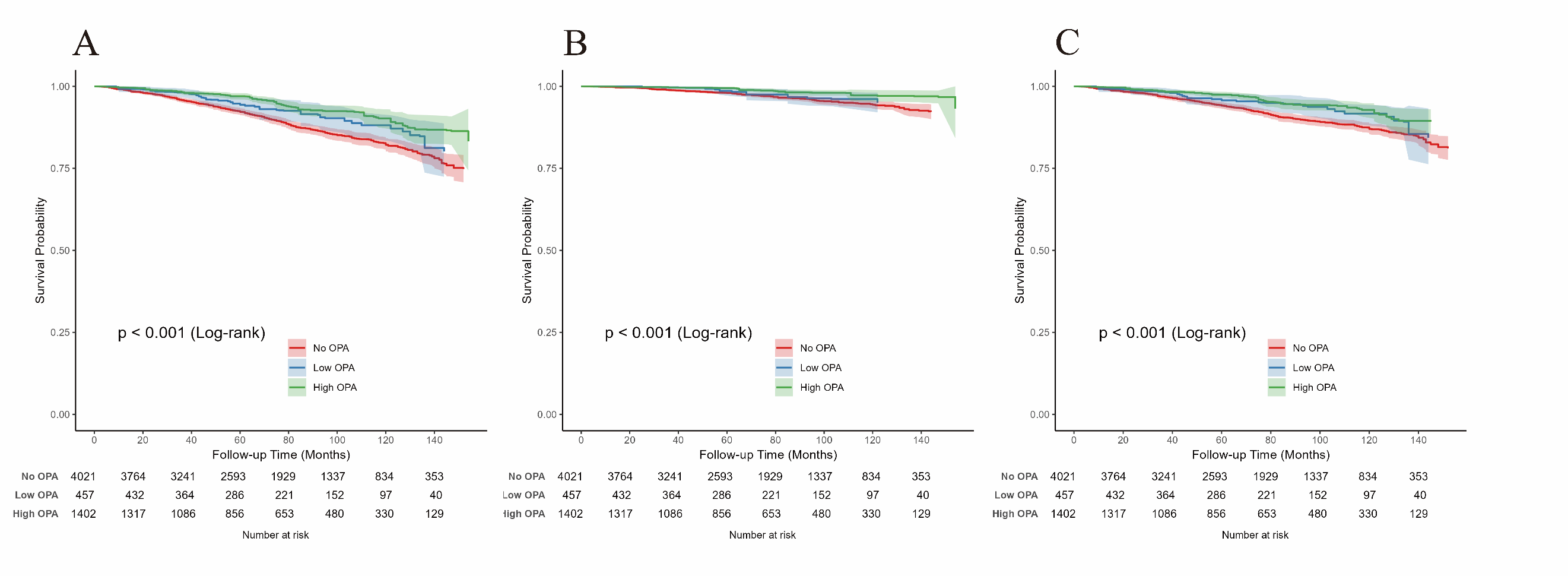


**Figure S3. Kaplan–Meier survival curves for all-cause, CVD, and non-CVD mortality by occupational physical activity levels and quartiles.**

(A): All-cause mortality, (B): CVD mortality, (C): non-CVD mortality.

All survival curves were weighted using NHANES sampling weights and accounted for complex survey design.

Abbreviations: NHANES: National Health and Nutrition Examination Survey; OPA: Occupational physical activity; CVD: Cardiovascular disease.


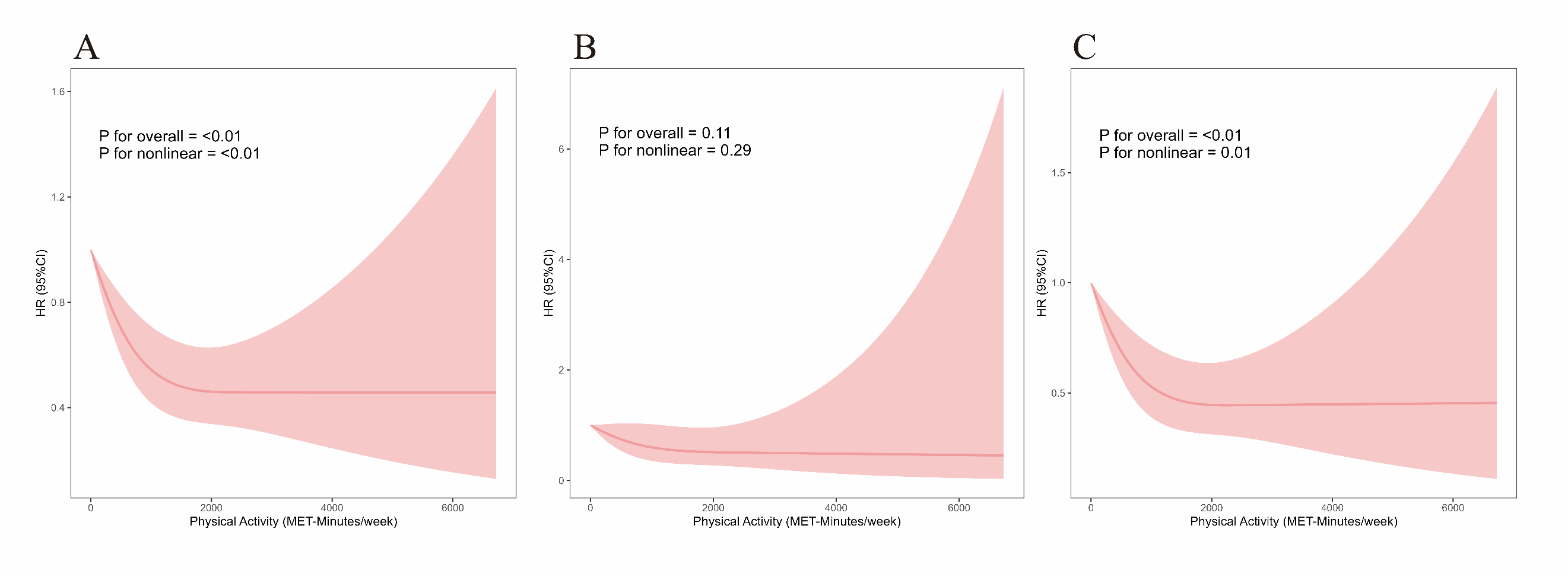


**Figure S4. Associations between leisure-time physical activity and mortality risk using restricted cubic spline models.**

(A): All-cause mortality, (B): CVD mortality, (C): non-CVD mortality.

All survival curves were weighted using NHANES sampling weights and accounted for complex survey design and adjusted for age, age at menopause, race, BMI, PIR, education level, marital status, alcohol consumption, smoking status, coronary heart disease, congestive heart failure, stroke, diabetes, hypertension, hyperlipidemia, MHT, depression, and oophorectomy history.

Abbreviations: NHANES: National Health and Nutrition Examination Survey; HR: Hazard ratio; 95% CI: 95% Confidence interval; MET: Metabolic equivalent of task; CVD: Cardiovascular disease; BMI: Body mass index; PIR: Poverty-to-income ratio; MHT: Menopausal hormone therapy.

**
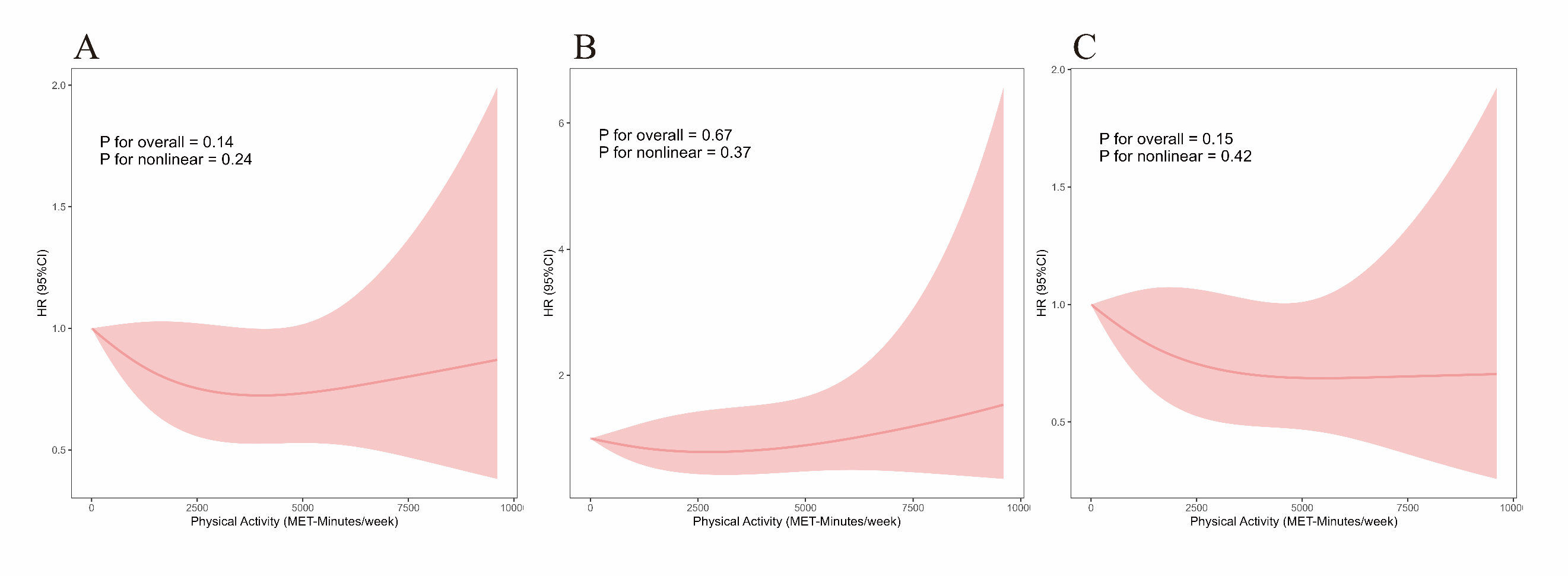
**

**Figure S5. Associations between occupational physical activity and mortality risk using restricted cubic spline models.**

(A): All-cause mortality, (B): CVD mortality, (C): non-CVD mortality.

All survival curves were weighted using NHANES sampling weights and accounted for complex survey design and adjusted for age, age at menopause, race, BMI, PIR, education level, marital status, alcohol consumption, smoking status, coronary heart disease, congestive heart failure, stroke, diabetes, hypertension, hyperlipidemia, MHT, depression, and oophorectomy history.

Abbreviations: NHANES: National Health and Nutrition Examination Survey; HR: Hazard ratio; 95% CI: 95% Confidence interval; MET: Metabolic equivalent of task; CVD: Cardiovascular disease; BMI: Body mass index; PIR: Poverty-to-income ratio; MHT: Menopausal hormone therapy.
